# Wider-community Segregation and the Effect of Neighbourhood Ethnic Diversity on Social Capital: An Investigation into Intra-Neighbourhood Trust in Great Britain and London

**DOI:** 10.1177/0038038516641867

**Published:** 2016-05-10

**Authors:** James Laurence

**Affiliations:** The University of Manchester, UK

**Keywords:** ethnic diversity, inter-ethnic relations, multi-level analysis, segregation and integration, social capital, trust, UK and London

## Abstract

Extensive research has demonstrated that neighbourhood ethnic diversity is negatively associated with intra-neighbourhood social capital. This study explores the role of segregation and integration in this relationship. To do so it applies three-level hierarchical linear models to two sets of data from across Great Britain and within London, and examines how segregation across the wider-community in which a neighbourhood is *nested* impacts trust amongst neighbours. This study replicates the increasingly ubiquitous finding that neighbourhood diversity is negatively associated with neighbour-trust. However, we demonstrate that this relationship is highly dependent on the level of segregation across the wider-community in which a neighbourhood is nested. Increasing neighbourhood diversity only negatively impacts neighbour-trust when nested in more segregated wider-communities. Individuals living in diverse neighbourhoods nested within integrated wider-communities experience no trust-penalty. These findings show that segregation plays a critical role in the neighbourhood diversity/trust relationship, and that its absence from the literature biases our understanding of how ethnic diversity affects social cohesion.

## Introduction

The question of how ethnic diversity affects societal cohesion has received considerable attention, stemming from studies suggesting that diversity can harm social capital (Putnam, 2007). In particular, concern has arisen due to the consistency of findings that higher neighbourhood diversity is associated with lower social capital *within* neighbourhoods (Van der Meer and Tolsma, 2014). This work emerged alongside (and later served as evidence for) claims within the public/political sphere that ethnic diversity threatens social solidarity (Goodhart, 2013). Given the historically high ethnic diversity across Europe, understanding the impacts of diversity is crucial. However, issues exist with the current literature.

While attention has focused on how the *level* of ethnic diversity affects social capital, far less has been paid to segregation. This is a substantial omission given segregation is ‘arguably the “structural linchpin” of … race relationsʼ (Bobo and Zubrinsky, 1996: 883). Studies have begun exploring how segregation across cities affects generalised trust (Rothwell, 2012; Uslaner, 2012); however, almost no research has examined the role of segregation in the relationship between *neighbourhood* diversity and within*-neighbourhood* social capital (although see Sturgis et al., 2013). Given diversity’s apparent negative effects appear most consistent within neighbourhoods, such an omission requires addressing.

This article examines the role of segregation in the relationship between neighbourhood ethnic diversity and neighbourhood social capital. In the literature, segregation is applied as either an alternative measure to diversity (Semyonov and Glikman, 2009) or as a complementary measure, alongside diversity, measured at the same contextual level (Uslaner, 2012). Drawing on theories of *nestedness*, in which smaller units are ‘integrally linked to, and dependent on, a larger wholeʼ, we generate a framework positing that it will be segregation in the wider-community in which a neighbourhood is *nested* that will matter for *within*-neighbourhood social capital (Sampson, 2012: 56).

Using Great Britain as a case study, we confirm the oft-observed negative association between neighbourhood diversity and neighbour-trust. However, this relationship is highly dependent on the level of segregation in the wider-community: it is diverse neighbourhoods nested in segregated wider-communities which exhibit lower trust. Individuals in diverse neighbourhoods nested within integrated wider-communities do not report lower social capital compared to their counterparts in homogeneous areas. We observe this relationship both across Great Britain and within the diverse city of London alone. These findings have important implications for the diversity/cohesion debate, and the contextual-effects literature more generally.

## Theoretical Framework and Evidence-base

### Ethnic Diversity and Social Capital

We begin by briefly summarising the theoretical-framework applied in studies examining diversity and social capital (for fuller discussions see Van der Meer and Tolsma, 2014). One set of theories predicts that increasing ethnic out-group size will undermine social capital. Firstly, group-threat theories posit that ‘a superordinate group [e.g. white British] becomes more racially hostile as the size of a proximate subordinate group increases, which putatively threatens the former’s economic and social privilegeʼ (Blalock, 1967; Oliver and Wong, 2003: 568). Secondly, experimental research on implicit-biases suggests that cohesion is more likely to develop within ethnic groups, as ‘racial differences [can] discourage the reliance on the behaviour of one’s neighbours … reducing levels of interpersonal trustʼ (Messick and Kramer, 2001; Stolle et al., 2008: 59). Similarly, studies drawing on concepts of ‘homophily’ suggest individuals in heterogeneous communities may ‘interact less frequently, which in turn leads to lower levels of interpersonal trustʼ (Letki, 2008: 104).

Conversely, the contact hypothesis posits that diverse environments may increase inter-group trust and cohesion (Allport, 1954). Diversity provides opportunities for interaction with ethnic out-groups that may be ‘conducive to the … kind of generalised trust that expands to include both members of the in- and out-groupʼ (Stolle et al., 2008: 59; Uslaner, 2012). Such ‘bridging capital’ may cultivate a more encompassing trust between groups in which broader identities and social solidarity are formed (Putnam, 2007; Uslaner, 2012).

A considerable volume of research has tested this framework, exploring the association between contextual ethnic diversity and social capital. This work stems largely from Putnam’s (2007) article demonstrating that residents of diverse communities report depleted social capital across a range of indicators. This is posited to emerge from diversity cultivating weaker inter-group *and* intra-group trust. Based on this, Putnam proposes the ‘constrict’ hypothesis: that diversity undermines social capital by eroding relations between *all* residents (both within and between ethnic-groups).

On the whole, the evidence-base presents mixed support for the ‘constrict’ hypothesis (see Van der Meer and Tolsma, 2014 for a review). However, in spite of the lack of evidence for Putnam’s (2007) general claim, reviews demonstrate that ‘there is consistent support for the constrict claim for aspects of social cohesion that are spatially bounded to neighbourhoodsʼ (Van der Meer and Tolsma, 2014: 459). This has been found across numerous countries and even demonstrated longitudinally (Laurence and Bentley, 2016). A more refined hypothesis therefore is that neighbourhood diversity has a caustic effect on *intra-*neighbourhood social capital (Laurence, 2013). However, this emphasis on how the *amount* of diversity affects social capital has been criticised for its failure to account for *segregation* (Hooghe, 2007; Uslaner, 2012).

### Segregation and Social Capital

Compared to ethnic diversity, far less research has examined segregation’s impact on social capital. Recently, public-policy discourses have problematised ethnic residential segregation as a driver of ‘parallel lives’, in which minority groups live in isolation from wider-society, with little contact outside their own group, harming cohesion (Cantle, 2005). This approach largely focuses on patterns of segregation as expressions of cultural difference, emphasising self-segregation as a driver (Kalra and Kapoor, 2009). Yet, this overlooks the structural conditions through which segregation also emerges and is sustained. Studies demonstrate that, alongside some degree of preferences for co-ethnic cohabitation (amongst both majority and minority populations), processes of prejudice and discrimination amongst the majority population, socio-economic inequalities and financial constraints, as well as natural population change and migration, play key roles in patterns of segregation (Bobo and Zubrinsky, 1996; Clark and Fossett, 2008; Krysan and Farley, 2002; Phillips, 2006; Simpson, 2004). Such processes often have distinct socio-political historical roots. For example, the segregation in Northern English mill towns (e.g. Oldham) is (in part) linked to deindustrialisation and the growing competition for economic/social resources, with more mobile Whites migrating away and discriminatory council housing policies and racial harassment leading to increased (enforced- and self-) segregation (Kalra and Kapoor, 2009; Kundnani, 2001). Segregation conceived as expressions of cultural difference, driven largely by self-segregation, fails to capture (both the historical and current) structural conditions which also contribute to patterns of segregation. This leads to persistent segregation being erroneously invoked to demonstrate failures of minority integration, when processes such as structural inequalities and discrimination also play critical roles (Kalra and Kapoor, 2009).

A range of processes are therefore implicated in structuring ethnic residential segregation. However, potentially, the *spatial patterns* of segregation that result from such processes could themselves, in turn, sustain or further foment processes of inter-group tensions in communities (Uslaner, 2012). In other words, the unequal distribution of groups across an area may itself trigger processes which cultivate (or prevent the neutralisation of) inter-group tensions.

Residential segregation captures some degree to which ethnic groups live in different areas (smaller geographic units) which collectively compose a larger geographic unit; for example, the distribution of groups across neighbourhoods in a city. It essentially expresses the degree to which groups are (un)equally distributed over a particular area. Importantly, segregation and diversity are not polar opposites: with no ethnic diversity there can be no segregation; however, equally diverse areas can be either highly integrated or highly segregated.

Segregation can be conceived across a number of dimensions, and measures are broadly grouped into exposure, evenness, centralisation, clustering and concentration (Massey and Denton, 1988). In this article we focus on the dimension of *evenness*; that is, how separately ethnic groups live from one another across a larger geographic unit. As we outline below, residential separateness may affect the psycho-social processes of contact/threat implicated in the ethnic diversity/social capital debate (Rothwell, 2012; Uslaner, 2012). We will illustrate the conception of segregation applied here with an example.

**Figure 1.**
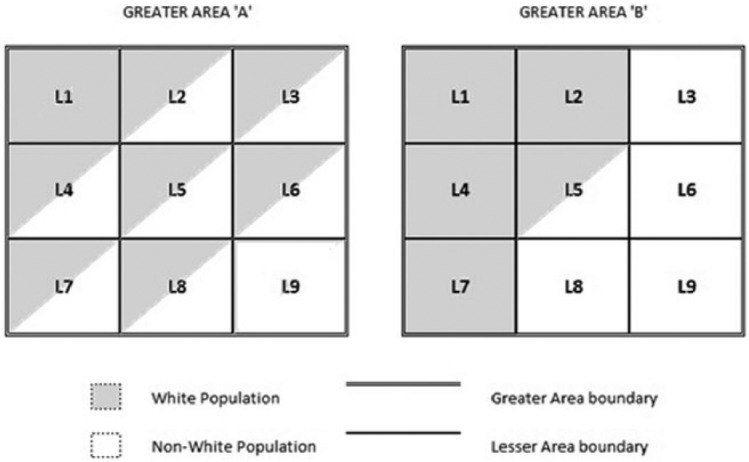
Diversity and segregation in two ‘Greater Areas’ composed of nine ‘Lesser Areas’.

As with most statistical measures of segregation, calculating evenness requires two contextual levels for operationalisation: a series of ‘Lesser Areas’ *nested* within a ‘Greater Area’. Figure 1 conveys a simplified view of two ‘Greater Areas’, each comprised of nine ‘Lesser Areas’. Both ‘Greater Area A’ and ‘B’ are equally diverse (50% White and 50% non-White); however, based on the distribution of ethnic groups across their ‘Lesser Areas’, ‘Greater Area A’ is largely integrated (evenly spread) while ‘Greater Area B’ is largely segregated (unevenly spread).

That *equally* diverse areas can be integrated or segregated suggests measuring diversity without accounting for segregation may be problematic. In Uslaner’s (2012) view, diversity harms social capital when there is little opportunity for contact between groups, i.e. in diverse, segregated environments (where groups largely live in separate areas), whilst there will be nominal exposure to one another (generating perceived threat), opportunities for inter-ethnic contact will be low, preserving negative out-group attitudes, undermining social capital (Semyonov and Glikman, 2009). In integrated areas increasing diversity may improve out-group relations, as a more even spread of groups throughout an area provides opportunities for out-group interactions, generating inter-group trust (Uslaner, 2012). Turning to Figure 1, if we took the approach of the current diversity literature, we would assume social capital will be equally low in both ‘Greater Area A’ and ‘Greater Area B’ given they are equally diverse. However, from a segregation perspective, residents of ‘Greater Area A’ would report comparatively higher social capital than residents of ‘B’ as the lower segregation of ‘A’ increases inter-ethnic contact opportunities.

Drawing on this framework, studies have explored how segregation affects social capital. Treating cities as the ‘Greater Area’, and neighbourhoods *nested* within cities as ‘Lesser Areas’, they examine the association between city-wide diversity, segregation and generalised indicators of social capital. Uslaner (2012) found that while city-level diversity undermined generalised trust, living in integrated cities could improve trust. Similarly, Rothwell (2012) demonstrated that segregation (not diversity) at the city-level harmed generalised trust. In this work, segregation appears more detrimental than diversity for social capital. However, it focuses on generalised trust, across large contextual-areas such as cities, in which diversity and segregation are measured at the same contextual-level. As outlined, the most consistent negative relationship is between neighbourhood ethnic diversity and social capital *within* neighbourhoods in particular. This study aims to examine the role of segregation in this neighbourhood diversity/neighbour-trust relationship.

### Segregation and Diversity: A Reformulation for Neighbourhood Analysis

There are two ways in which segregation may matter in the study of neighbourhood diversity/neighbour-trust: firstly, via segregation *within* neighbourhoods; and secondly, via segregation across the wider-community in which a neighbourhood is nested.

In testing how diversity amongst neighbours is associated with trust between them, diversity needs to be measured at as close an approximation of one’s neighbours as possible (defined in our current data as an individual’s ‘immediate neighbourhood, i.e. your street/block’). One approach to examining the role of segregation would be to measure levels of segregation *within* the immediate neighbourhood, i.e. at the same contextual-level as neighbourhood diversity (cf. Uslaner, 2012). Segregation can exist *within* a street/block, e.g. if groups populate different ends/sides of a street. However, segregation across these small areas might not lead to substantial differences in opportunities for inter-ethnic contact due to spatial divisions alone; close proximity should still generate inter-group interactions. Therefore, when analysing the effect of immediate-neighbourhood diversity on neighbour-trust, a measure of *within* immediate-neighbourhood segregation might add little additional information on the differential opportunities for inter-ethnic interaction that Uslaner (2012) suggests is necessary to properly test diversity’s effect on social capital. Ultimately, however, this remains an empirical matter and testing it with current data is difficult.^1^

Another way in which segregation may matter for neighbour-trust is through levels of segregation across the wider-community in which a neighbourhood is *nested*. Returning to Figure 1, let us assume that ‘Lesser Areas’ represent neighbourhoods and ‘Greater Areas’ represent the wider-communities in which neighbourhoods are nested. As we can see, each neighbourhood nested within a wider-community has its own level of diversity. The current diversity literature assumes that residents of equally diverse neighbourhoods in both wider-communities would report equally low neighbour-trust. However, as Sampson (2012: 54) points out, ‘neighborhoods are nested within successively larger communitiesʼ and ‘the local neighborhood is integrally linked to, and dependent on, a larger wholeʼ. We suggest that, after accounting for how diverse neighbours are in the immediate neighbourhood (i.e. the ‘Lesser Area’), segregation across the wider-community (i.e. the ‘Greater Area’) in which the neighbourhood is *nested* could have an additional effect on trust between those same neighbours. Below we outline a framework for this.

### Wider-community Segregation, Neighbourhood Ethnic Diversity and Neighbour-trust

**Figure 2.**
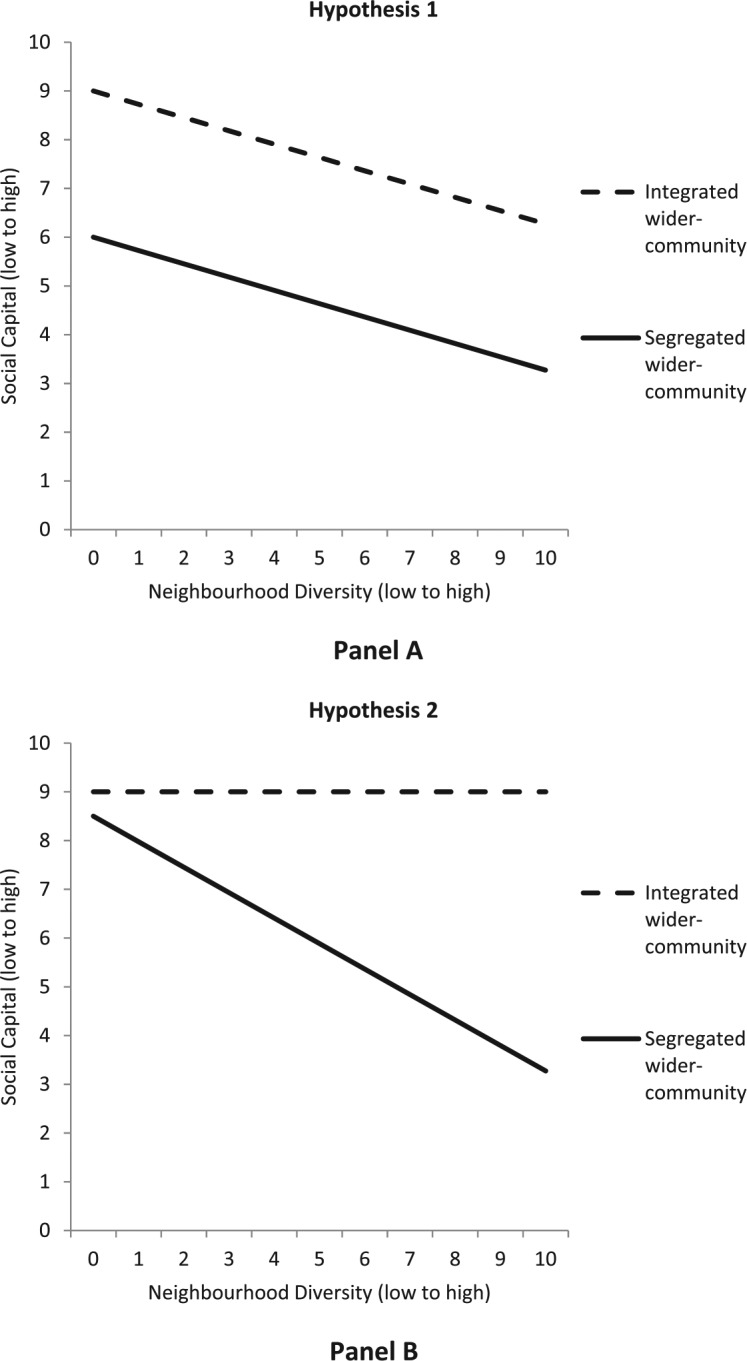
Simplified conceptual models of Hypothesis 1 and 2.

Our first hypothesis (Hypothesis 1) is that: after accounting for the negative effect of neighbourhood diversity, wider-community segregation will exert an additional *direct* negative effect on neighbour-trust. Figure 2 (Panel A) shows a simplified version of this hypothesis. We outline the reasoning below.

Firstly, higher segregation across wider-communities may generate greater perceived-threat.^2^ In segregated wider-communities, out-groups will be more spatially concentrated, which may ‘exaggerate the degree of difference between groupsʼ, making the out-group ‘seem larger and more menacing than it isʼ (Allport, 1954: 18–19). This may render out-group ‘difference … more visible, and … assimilation seem more uncertainʼ, especially if areas take on stronger out-group characteristics; for example, shops, schools and community centres serving particular out-group cultural needs may foster a sense of exclusion/alienation (Biggs and Knauss, 2012: 635). Relatedly, as segregated wider-communities contain denser out-group neighbourhoods, allocations of government funds to such areas may foster perceptions of inter-ethnic competition (while the absence of such neighbourhoods in integrated wider-communities precludes such claims gaining traction) (Rhodes, 2009). Insofar as higher group-threat can undermine neighbour-trust (Schmid et al., 2014), segregation *across* the wider-community may have a direct negative effect on neighbour-trust via perceived-threat.

An alternative hypothesis is that, given we are focusing on trust in one’s neighbours in particular, any impact of living in a segregated wider-community may not affect all individuals equally. Instead, how far wider-community segregation impacts an individual’s neighbour-trust may depend on the ethnic composition of one’s neighbours. Our second hypothesis (Hypothesis 2) is that: wider-community segregation will *moderate* the association between neighbourhood diversity and neighbour-trust. Individuals in homogeneous neighbourhoods will report equally high levels of neighbour-trust, regardless of wider-community segregation. Individuals in diverse neighbourhoods nested within segregated wider-communities will report low trust. However, individuals in diverse neighbourhoods nested within integrated wider-communities will report high neighbour-trust (see Figure 2 (Panel B)). We outline our reasoning below.

Firstly, if feelings of perceived-threat are heightened amongst residents of segregated wider-communities then where out-groups *do* cohabit in diverse neighbourhoods, trust between those neighbours will likely be lower compared to equally diverse neighbourhoods in integrated wider-communities (where perceived-threat is lower). However, in homogeneous neighbourhoods, even under conditions of higher perceived-threat in segregated wider-communities, neighbour-trust should be unaffected given there are no out-group neighbours to distrust.

Secondly, processes of threat, perceived-difference and assimilation-uncertainty across segregated wider-communities could also affect elements of inter-group contact *within* neighbourhoods. In the literature, equally diverse neighbourhoods are believed to lead to equal amounts of inter-group contact, and that this contact reduces prejudice. The segregation literature thus suggests contact will be lower across wider-communities that are segregated as more homogeneous neighbourhoods reduce contact-opportunities (Uslaner, 2012). However, the likelihood of inter-group contact occurring in equally diverse neighbourhoods may differ depending on whether the neighbourhood is nested within a segregated/integrated wider-community. Perceiving greater group differences, and experiencing threat, uncertainty and anxiety towards out-groups, can inhibit likelihoods of cross-group contact (Allport, 1954; Schmid et al., 2014). If these feelings are higher across segregated wider-communities, individuals in diverse neighbourhoods may experience comparatively less neighbour out-group contact than in equally diverse neighbourhoods within integrated wider-communities.

This is important as studies show that inter-group ties moderate the association between neighbourhood diversity and neighbour-trust: it is only residents of diverse neighbourhoods who do *not* possess out-group ties that report lower neighbour-trust; residents of diverse neighbourhoods with ties *and* residents of homogeneous neighbourhoods (with or without ties) report higher neighbour-trust (Laurence, 2011; Stolle et al., 2008). If individuals in diverse neighbourhoods in segregated wider-communities are less likely to form out-group ties, then living in a diverse neighbourhood may have a stronger negative effect on neighbour-trust in these wider-communities.

A third possibility concerns assumptions that all contact has an equal likelihood of rescinding prejudice; superficial, unequal, negative, involuntary contact may do little (Allport, 1954). While neighbourhood diversity increases opportunities for neighbourhood contact, segregation in the wider-community may result in more segregated organisational-foci in the wider-community (e.g. schools, workplaces, social activities, religious institutions may be more ethnically homogeneous). One possibility is that diverse neighbourhoods alone are not sufficient to build the kind of inter-ethnic ties necessary for prejudice reduction. More sustained, meaningful interaction in organisational-foci may be needed, or may be more conducive to building inter-group trust, compared to more casual neighbourhood interactions. Individuals in segregated wider-communities may therefore experience less efficacious out-group contact. If individuals in diverse neighbourhoods *without* such ‘meaningful’ contact trust their out-group neighbours less, then increasing neighbourhood diversity in segregated wider-communities may again have a stronger negative impact on neighbour-trust.

### Summary

This study investigates the role of segregation across wider-communities (as conceived as evenness/separateness) in the neighbourhood-diversity/neighbour-trust relationship. As outlined, statistical measures of segregation are commonly captured across a ‘Greater Area’ geographical-unit, containing a series of ‘Lesser Areas’ *nested* within, each with distinct levels of diversity. Previous studies tend to view segregation as an alternative measure to diversity (Hooghe, 2007; Semyonov and Glikman, 2009), or a complementary measure at the same geographical scale (Rothwell, 2012; Uslaner, 2012). We suggest that, for trust *within*-neighbourhoods, it will be living in diverse ‘Lesser Areas’ in ‘Greater Areas’ where most groups live apart (i.e. that are segregated) that may be particularly problematic.

## Data and Methodology

### Datasets

Our main analysis will use the Great Britain 2000–01 General Household Survey (GHS) (collected between April 2000–March 2001). The GHS achieved a response rate of 67%, applying a multi-stage sampling design (households sampled within Wards). The second dataset applied is the Metropolitan Police Public Attitudes Survey (METPAS). The METPAS constitutes a sample of individuals living in London alone (response rate over 60%) which we use to stringently test our hypotheses. Data from the 06–07 and 07–08 waves were combined into a single data set to increase the *n within* our level 2/level 3 clusters for greater model robustness.^3^ The METPAS used a multi-stage design, sampling households within 32 London Local Authorities.

The GHS data precedes a number of significant events, such as 9/11 and 2004 EU enlargement, which may affect the salience of issues of ethnicity/immigration and therefore the processes being examined. The GHS thus provides opportunities to test our hypotheses outside of such a context, while the METPAS data occurs after such events. Extensive data-checking and -cleaning was undertaken by the Office for National Statistics (GHS) and the METPAS research team/BMG survey-group (METPAS) before release (see data technical reports for details). Additional testing/cleaning included recoding non-substantive responses (e.g. ‘don’t know’) as missing, excluding cases with missing data on our applied variables, testing model assumptions and examining potential outlier-bias in our analysis.

### Key Dependent Variable

Our dependent variable is ‘trust in neighbours’. When answering this question individuals are explicitly instructed to think of their ‘more immediate neighbourhood (by which I mean your street or block)’ and then probed as to whether they ‘trust … most, many, a few, or none of the people in your neighbourhood’. This measure mirrors the key outcome in Putnam’s (2007) models as well as much of the literature.

### Key Independent Variables

#### Immediate Neighbourhood Diversity

The UK census provides data at a range of spatial levels based on Output-Area classifications. These are small-scale area-levels designed to be approximately the same size/shape, and constrained by obvious boundaries (e.g. major roads), forming pedestrian-friendly areas conforming to residential neighbourhoods. As our dependent variable explicitly asks respondents to think about trust in their ‘immediate neighbourhood (by which I mean your street or block)’ we measure diversity at the smallest available area: the output-area (OA) (average 250 residents). This is the closest approximation of the ‘immediate neighbourhood’. Ethnic diversity is measured using the Simpson’s Index of Fractionalisation (SDI):

$$Ethnic\;Diversity_{j}=1-\underset{k}{\sum }S_{kj}^{2}$$

where *j* stands for the neighbourhood area and *k* for the ethnic group.

Census ethnicity categories are grouped into 10 sub-groups, including: ‘White British’, ‘White Other’, ‘Pakistani’, ‘Bangladeshi’, ‘Indian’, ‘Asian Other/Mixed’, ‘Black Caribbean/Mixed/Black Other’, ‘Black African/Mixed’, ‘Chinese’, and ‘Other/Mixed’.

### Wider-community Segregation

To measure residential segregation we apply the Index of Dissimilarity (ID):

$$\frac{1}{2}\sum_{i=1}^{b_{i}}\frac{b_{i}}{B}-\frac{w_{i}}{W}$$

*b_i_* = the population of ‘*non-White British*’ in the *ith* area, i.e. the ‘Lesser Area’

*B* = the total population of ‘*non-White British*’ in the ‘Greater Area’

*w_i_* = the population of ‘*White British*’ in the *ith* area, i.e. the ‘Lesser Area’

*W* = the total population of ‘*White British*’ in the ‘Greater Area’

The ID captures the *evenness* of the distribution of two populations across a series of ‘Lesser Areas’ that form components of a ‘Greater Area’. The ID is the most frequently applied measure of segregation and provides intuitive interpretation: values (from 0 to 1) represent the proportion of a group required to move to generate a completely even distribution of groups across an area. This measure is useful given it is largely independent of the size of groups used in its composition (as other measures can be highly correlated with diversity).^4^ The closest UK geographic-scale at which to measure the wider-community to that used in previous segregation studies (e.g. Rothwell, 2012; Uslaner, 2012) is the Local Authority-level (*mean*: 120,000 people, containing around 650 OAs). The Local Authority (LA) thus forms the ‘Greater Area’ across which we measure the ID, while OAs form the ‘Lesser Areas’ nested within.

To measure the ID, we compare ‘White British’ (majority group) and ‘non-White British’. Firstly, as the bulk of our GHS sample (95%) is made up of White British, their segregation first needs to be captured. Secondly, the theory behind studying diversity in the literature is that, theoretically, exposure to all ethnic out-groups exerts an equally negative effect on trust (although studies have demonstrated differential effects (Laurence, 2011)). Based on this, which group one is segregated from may matter less than their overall level of segregation from all out-groups (although further analysis should test whether *who* groups are segregated from matters). We also experimented with multi-group measures of segregation, which capture segregation between all groups; however, this returned highly similar findings, and for intuitiveness of interpretation, and comparability with the literature, we report results using the ID.^5^

### Covariates

We adjust for neighbourhood (OA) level characteristics: residential turnover (the inflow and outflow of persons in an area) per 1000 people from 2000–2001; whether the neighbourhood is in a town or urban (compared to rural) area; population density; % aged 65+; and an Index of Crime.^6^ We also include two indices of disadvantage, derived from a factor analysis of: % unemployed; % female single-parent households; % ‘long-term unemployed/never worked’; % social housing; % with no qualifications; and % elementary/process occupations. Table 1 shows these indicators load onto two distinct measures of disadvantage: ‘resource-vulnerability’ (Factor 1) and ‘status-vulnerability’ (Factor 2). Our community-level data is predominantly taken from the 2001 UK Census. At the individual-level we adjust for: length of time lived in neighbourhood, age, gender, education, Socio-Economic Class, ethnicity, economic-status, marital status, children in household, country-of-birth, and housing tenure. We also include a dummy variable capturing survey year in the METPAS analysis.

**Table 1.** Factor analysis of community disadvantage variables.

Variable	Factor 1	Factor 2
% Unemployed (Economically active)	0.86	0.24
% Social housing	0.73	0.36
% Long-term unemployed and never worked	0.87	0.18
% Female lone-parent	0.72	0.29
% No qualifications	0.39	0.73
% Elementary/Process occupations	0.14	0.76
Eigenvalue	2.81	1.44

*Notes*: Orthogonal Varimax Rotation (Kaiser On).

### Modelling Methods

Our data structure contains three levels: individual (level-1), immediate-neighbourhood (level-2), and wider-community (level-3).^7^ This nested structure requires hierarchical modelling techniques (Snijders and Bosker, 1999). A key hypothesis is that the slope of diversity at level-2 may vary depending on segregation at level-3. We thus apply random-coefficient models allowing for this and ordered logistic regression to account for the ordinal structure of ‘trust in neighbours’.

## Results

### Immediate Neighbourhood Diversity, Wider-community Segregation and Neighbour-trust in Great Britain

We begin by exploring the association between neighbourhood ethnic diversity and neighbour-trust. Model 1 (Table 2) demonstrates that immediate neighbourhood ethnic diversity is negatively associated with neighbour-trust, replicating the key finding of the current literature. However, both resource- and status-vulnerability have stronger negative associations with neighbour-trust. Furthermore, the strongest negative association (almost twice that of diversity) is living in an urban compared to rural neighbourhood. At the individual-level, higher socio-economic class, homeownership and higher qualifications predict greater neighbour-trust, alongside age and length of time in the community. Furthermore, Black British individuals and ‘other ethnic groups’ report lower neighbour-trust, compared to White British. We also observe a stronger negative association between ethnic diversity and neighbour-trust amongst lower socio-economic classes (especially the ‘long-term unemployed/never-worked’), and those with lower qualifications (full results available on request).

**Table 2.** Wider-community segregation, neighbourhood ethnic diversity and neighbour-trust across Great Britain; GHS data.

	Model 1	Model 2	Model 3
Dependent variable:	Neighbour trust	Neighbour trust	Neighbour trust
Sample:	All	All	All
*Neighbourhood (OA) level (level 2)*:			
Density	−0.058*	−0.057*	−0.066*
	(0.028)	(0.028)	(0.028)
Turnover	−0.028	−0.025	−0.020
	(0.032)	(0.032)	(0.032)
% age 65+	0.102***	0.102***	0.101***
	(0.029)	(0.029)	(0.029)
Town (cf. village)	−0.109**	−0.109**	−0.108**
	(0.036)	(0.036)	(0.036)
Urban (cf. village)	−0.264***	−0.265***	−0.268***
	(0.041)	(0.041)	(0.041)
Resource-vulnerability	−0.142**	−0.140**	−0.144**
	(0.050)	(0.050)	(0.050)
Status-vulnerability	−0.216***	−0.224***	−0.209***
	(0.047)	(0.048)	(0.049)
IMD crime	−0.093*	−0.099**	−0.107**
	(0.036)	(0.037)	(0.037)
Diversity	−0.160***	−0.165***	−0.126*
	(0.048)	(0.049)	(0.052)
*Wider-community (LA) level (level 3)*:			
Segregation		0.031	0.026
		(0.035)	(0.035)
*OA-level x LA-level (level 2 x level 3)*:			
Diversity * Segregation			−0.082**
			(0.033)
*N*	6072	6072	6072
*N* (OAs)	5265	5265	5265
*N* (LAs)	270	270	270

*Notes*: ***p < 0.001; **p < 0.01; *p < 0.05; *p* < 0.1. Standardised coefficients; models include individual-level covariates: gender, social class, time in area, ethnicity, marital status, tenure, employment status, UK-born, children in household, age.

We next test whether Local Authority segregation has a direct impact on neighbour-trust (Hypothesis 1). Model 2 runs a three-level model (individuals *nested* in OAs *nested* in Local Authorities) and demonstrates that Local Authority segregation has no direct association with neighbour-trust. This suggests Hypothesis 1 is not supported.

To test whether wider-community segregation *moderates* the association between neighbourhood diversity and neighbour-trust (Hypothesis 2) we include a cross-level interaction term between immediate neighbourhood (OA) ethnic diversity (level 2) and Local Authority segregation (level 3). This interaction is negative and significant, indicating that increasing neighbourhood diversity has a stronger negative association with neighbour-trust if neighbourhoods are *nested* within more segregated Local Authorities (Model 3). This begins to provide evidence for Hypothesis 2. This association persists when tested amongst our White sample alone.^8^ However, although our non-White sample is small (*n* = 323), the interaction is also observed within this group.^9^

**Figure 3.**
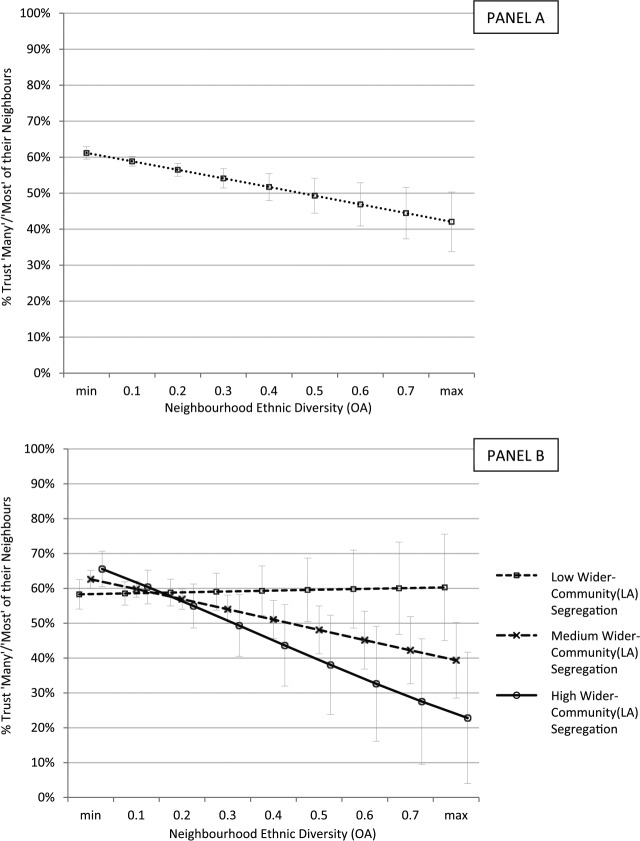
Predicted neighbour-trust by neighbourhood ethnic diversity (OA) *and* wider-community (LA) White-GB v non-White-GB segregation; Great Britain (GHS data). *Notes*: other variables set to their mean; min. diversity = 0; max. diversity = 0.85; low district segregation (0-.3 ID); medium district segregation (.3-.6 ID); high district segregation (.6-1 ID).

To explore how this interaction manifests itself across our data we created a series of predicted neighbour-trust scores. Firstly, Figure 3 (Panel A) displays the predicted percent of individuals that ‘trust many/most’ of their neighbours at nine levels of neighbourhood diversity, derived from Model 1 (Table 2). Secondly, Figure 3 (Panel B) displays predicted trust at nine levels of ethnic diversity but sub-divided across three grouped levels of Local Authority segregation (based on predicted scores derived from Model 3). To help substantive interpretation of these findings, we show the average effect of neighbourhood diversity across ‘low’, ‘medium’ and ‘high’ levels of segregation, based on Massey and Denton’s (1993) categorisation of low (< .3 ID), medium (> .3 ID and < .6 ID) and high (> .6 ID) segregation.^10^

Figure 3 (Panel A) shows the direct effect of diversity: residents in highly diverse neighbourhoods (max: 0.85 SDI) are around 18 percentage points less likely to ‘trust many/most’ of their neighbours, compared to individuals in homogeneous neighbourhoods. However, Figure 3 (Panel B) shows how dependent this association is on wider-community segregation: in low segregated Local Authorities, individuals living in highly diverse neighbourhoods see a (non-significant) *increase* in trust, compared to residents of homogeneous neighbourhoods. However, individuals living in highly diverse neighbourhoods *nested* within segregated Local Authorities (> .6 ID) see a (significant) 32 to 40 percentage point reduction in trust, compared to individuals in homogeneous neighbourhoods *nested* in either integrated or segregated Local Authorities respectively. Crucially, they also report a 38 percentage point reduction in trust compared to residents of *equally diverse* neighbourhoods *nested* within integrated Local Authorities (< .3 ID). Figure 3 thus provides evidence for Hypothesis 2: it is only individuals living in wider-communities with medium, and especially high, segregation whose neighbour-trust declines with increasing neighbourhood diversity.^11^

These findings demonstrate that only diverse neighbourhoods *nested* within more segregated wider-communities appear to exhibit depressed neighbour-trust. However, counter-claims remain. Firstly, neighbourhood (OA) level diversity is correlated with wider-community (LA) level diversity (*r* = .77). We could be measuring the effects of living in a diverse *and* segregated wider-community, not a diverse neighbourhood *nested within* a segregated wider-community. However, there is no significant interaction between LA-level diversity and LA-level segregation for neighbour-trust (*coef.*: −0.05 n/s). Secondly, if we include our OA-level diversity/LA-level segregation interaction-term alongside an LA-level diversity/LA-level segregation interaction-term, the former remains significant (*coef*: 0.082*). Another possibility is that LA-level segregation is acting as a proxy for LA-level diversity. However, including LA-level diversity in the model (which is not significant after accounting for neighbourhood (OA) diversity), as well as testing an interaction term between LA-level diversity and OA-level diversity, does nothing to diminish our key interaction term. A further possibility is that LA-level segregation is acting as a proxy for other LA-level characteristics (e.g. disadvantage), and our models are driven by OA-level diversity exhibiting stronger negative effects in more disadvantaged LAs, for example. Firstly, we ran a model including all our current OA-level controls but at the LA-level as well. We also re-ran our full model (Model 3) but included interaction terms between OA-level diversity and these LA-level controls, e.g. an OA-level diversity/LA-level disadvantage interaction; our key finding remains.

In sum, these tests demonstrate that it is *wider-community segregation*, and an interaction with levels of *immediate neighbourhood diversity* nested within, which matters for neighbour-trust. However, another issue is that our index of wider-community segregation (the ID) is largely unaffected by the amount of diversity in the wider-community; equally high/low levels of the ID can be found in Local Authorities with either high or low levels of diversity. We investigated whether the moderating association between neighbourhood diversity/wider-community segregation was also dependent on the *amount* of diversity across the wider-community as well. Testing shows that even in Local Authorities with lower diversity, Local Authority segregation continues to condition the effect of neighbourhood diversity on trust. However, the strength of the moderating effect of Local Authority segregation on neighbourhood diversity becomes stronger in more diverse Local Authorities. Full results are omitted here but available on request.

### Robustness of Relationship: A London Study

To test the robustness of these findings we examine our hypotheses *within* London. While replicating our findings in another dataset provides a useful test, focusing on a single city helps reduce possible city-to-city unobserved heterogeneity that may drive our Great Britain analysis. Furthermore, London is a historically diverse, integrated urban-area, with significant past immigration and comparatively higher tolerance (Sturgis et al., 2013). Therefore, London should form a more stringent test-bed.

We thus turn to our second dataset: METPAS. Firstly, the LSOA (not OA) is the smallest spatial-unit respondents could be linked to. Therefore, the neighbourhood is measured at the LSOA-level. Thus, Local Authority segregation is measured using the LSOA (not OA) as the ‘Lesser Area’. Neighbour-trust is measured by the question: ‘to what extent do you agree or disagree … people in this neighbourhood can be trusted’ (‘strongly agree’ to ‘strongly disagree’). This differs somewhat from the GHS question, potentially capturing the quality of the relationships versus the quantity. However, compared to the GHS data, the METPAS contains a larger non-White British sample (38% non-White British); thus, we can generate more robust estimates of ethnic diversity’s effects.^12^

Table 3 shows a series of random-coefficient, HLMs (three-level models of individuals *nested* in LSOAs *nested* in Local Authorities). Model 1 shows that, unlike across Great Britain as a whole, diversity has a negative but non-significant linear association with neighbour-trust. Thus, living in more diverse neighbourhoods alone does not appear to impact neighbour-trust across London. Model 2 demonstrates that there is no significant direct association between wider-community segregation and neighbour-trust. However, Model 3 shows that, as previously found across Great Britain, neighbourhood ethnic diversity is significantly moderated by segregation across Local Authorities.

**Table 3.** Wider-community segregation, neighbourhood ethnic diversity and neighbour-trust in London; METPAS data.

	Model 1	Model 2	Model 3
Dependent variable:	Neighbour trust	Neighbour trust	Neighbour trust
Sample:	All people	All people	All people
*Neighbourhood (LSOA) level (level 2)*:			
Density	−0.021	−0.021	−0.022
	(0.024)	(0.024)	(0.024)
% in same community 1 year ago	0.046*	0.040+	0.040+
	(0.023)	(0.024)	(0.024)
% age 65+	0.056*	0.056*	0.051*
	(0.023)	(0.023)	(0.023)
Resource-vulnerability	0.073+	0.072	0.059
	(0.044)	(0.044)	(0.044)
Status-vulnerability	−0.089*	−0.088*	−0.099**
	(0.036)	(0.036)	(0.036)
IMD crime	−0.010	−0.010	−0.006
	(0.022)	(0.022)	(0.022)
Diversity	−0.06	−0.056	−0.037
	(0.036)	(0.036)	(0.038)
*Wider-community (LA) level (level 3)*:			
Segregation		−0.072	−0.076
		(0.119)	(0.116)
*LSOA-level x LA-level (level 2 x level 3)*:			
Diversity * Segregation			−0.079***
			(0.024)
*N*	16336	16336	16336
*N* (LSOAs)	4444	4444	4444
*N* (LAs)	32	32	32

*Notes*: ***p < 0.001; **p < 0.01; *p < 0.05; ^+^*p* < 0.1. Standardised coefficients; models include individual-level covariates: gender, social class, time in area, ethnicity, marital status, tenure, employment status, children in household, age.

**Figure 4.**
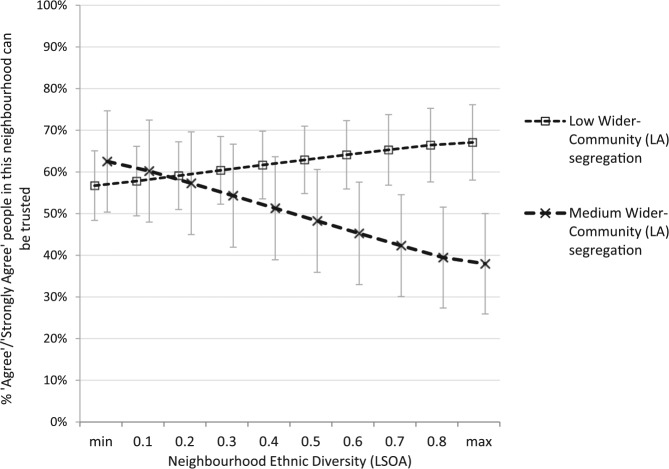
Predicted neighbour-trust by neighbourhood ethnic diversity (LSOA) *and* wider-community White-GB v non-White-GB segregation (LA); London (METPAS data). *Notes*: other variables set to their mean; min. diversity = 0.02; max. diversity = 0.87; low district segregation (0-.3 ID); medium district segregation (.3-.6 ID).

Based on Model 3 (Table 3) we again create a series of predicted trust scores across varying levels of neighbourhood diversity by Local Authority segregation, grouped into low (< .3 ID) and medium (> .3 ID and < .6 ID) segregation (Figure 4).^13^ Neighbour-trust in diverse neighbourhoods *nested* within more integrated Local Authorities is not significantly different from trust within homogeneous neighbourhoods. However, in diverse neighbourhoods *nested* within more segregated Local Authorities, residents are around 28 percentage points less likely to ‘agree/strongly agree’ people can be trusted, compared to those in equally diverse neighbourhoods *nested* within integrated Local Authorities.

In sum, increasing neighbourhood diversity is not, on average, associated with greater neighbour-distrust in London. However, we again observe that even within the diverse, integrated city of London, neighbourhood diversity has a stronger negative association with neighbour-trust in more segregated Local Authorities.^14^

### Selection Bias and Alternative Interpretations

These findings support our framework that processes generated *by* wider-community segregation may determine *when* neighbourhood diversity undermines neighbour-trust. However, patterns of diversity and segregation do not emerge randomly and various factors influence their emergence.

As outlined above, studies find that attitudes for in-/out-group cohabitation are one driver of segregation (Clark and Fossett, 2008; Krysan and Farley, 2002). Integrated wider-communities may develop (partly) in response to individuals sorting into/out-of areas based on prior attitudes. If such attitudes can drive patterns of segregation, then the non-effect of neighbourhood diversity in *integrated* wider-communities may be a consequence of such areas containing concentrations of individuals with preferences *for* diversity (experiencing no negative effect of neighbourhood diversity). If segregated wider-communities can develop from preferences for homogeneity, then where neighbourhood out-group exposure does occur in such wider-communities (e.g. at the boundaries between predominantly White and non-White areas, or where non-White groups are migrating into previously White neighbourhoods, or where more biased Whites have not yet (or cannot) migrate away) trust between neighbours may be lower.

Similarly, processes of discrimination, harassment and prejudice amongst majority populations can lead to enforced- or self-segregation amongst minorities (Bobo and Zubrinsky, 1996; Kundnani, 2001). Such processes also corrode neighbour-relations, especially in diverse neighbourhoods. Thus, these processes driving segregation could also drive lower neighbour-trust. Relatedly, segregation is also tied to structural socio-economic inequalities, affecting ability/opportunity to move, amongst minority and majority populations (Kalra and Kapoor, 2009). Often the most segregated areas have long histories of deprivation, disinvestment and disenfranchisement; as mentioned, these processes can have distinct socio-political, historical roots, e.g. in Northern English towns (Kundnani, 2001), or London (Glynn, 2010). Such structural inequalities, while contributing to segregation, may also lead to long-term social capital depressions.

In sum, our findings may not be driven (in part or in full) by wider-community segregation itself affecting processes of inter-ethnic contact/perceived-threat. Segregation may reflect other processes (e.g. discrimination, inequalities), with distinct historical roots, and it is these explaining the differential levels of trust across neighbourhood diversity between wider-communities. Unfortunately, we cannot fully address such issues here. However, this study still provides a vital comparative analysis to current cross-sectional analyses.

## Discussion and Conclusion

This article incorporates segregation into the question of how neighbourhood ethnic diversity affects neighbourhood social capital. Existing research demonstrates an ‘empirical regularityʼ: diverse neighbourhoods exhibit lower intra-community social capital (Van der Meer and Tolsma, 2014: 471). However, we demonstrate this oft-observed negative relationship is contingent on the level of wider-community segregation in which a neighbourhood is nested: only residents of segregated wider-communities experience a negative effect of increasing neighbourhood diversity. Individuals in diverse neighbourhoods *nested* within integrated wider-communities report similar social capital to residents of homogeneous neighbourhoods (*nested* in either integrated *or* segregated wider-communities).

We replicated our tests within London. Despite analysing this relationship in a single city, with a unique history and environment, the effect of neighbourhood diversity again appears stronger in more segregated wider-communities. Interestingly, neighbourhood diversity does not have a direct effect on neighbour-trust across London. However, on average, rates of Local Authority-level segregation are lower in London (ID 0.26) than across Great Britain (ID 0.43). When examining neighbourhood diversity’s effects *by* segregation within London, similar findings to our national analysis emerge. Lower average segregation in London could therefore account for the absence of a direct-effect of diversity.

This work contributes to the conceptualisation of segregation in diversity/social capital studies. Most segregation measures are calculated across a ‘Greater (geographic) Area’, containing a series of ‘Lesser Areas’ nested within, each with distinct levels of diversity. We suggest that, on top of the effects of segregation across higher geographical areas previously demonstrated (e.g. Uslaner, 2012), variations in the ethnic diversity of residents’ micro-scale environments (e.g. their immediate neighbourhoods) *within* these areas also matter. Interdependent processes occur at both the Greater- and Lesser-Area levels, leading to micro-scale differences in social capital outcomes (although this may be particularly important for intra-neighbourhood outcomes). However, such an approach may depend on the particular conception of segregation applied here: the *evenness* of ethnic-group distribution (operationalised using the Index of Dissimilarity). Further work could explore the relative salience of different dimensions of segregation (e.g. clustering, centralisation) for the diversity/social capital relationship. In addition, surveys with larger non-White British populations would allow for more robust tests of how these processes operate amongst minority populations.

This article suggests aspects of the theoretical framework applied to examine ethnic diversity and social capital may need re-evaluating. In particular, it questions the current operationalisation of the threat/contact hypothesis framework in diversity/social capital studies. Predominantly, threat and/or contact are posited to emerge solely from contextual diversity. However, processes of threat/contact may be conditioned by the characteristics of the wider-community (e.g. segregation) in which neighbourhoods are nested. These may determine *when* the size of out-group in a neighbourhood matters for social capital. Although, whether segregation itself is a cause/preservative of such processes, or actually a proxy for weaker inter-group relations already present, requires attention.

These findings also help manage an issue within ethnic diversity/segregation debates. While public-policy discourse problematises segregation and high minority-share areas, implicit within the cohesion/diversity debate is that heterogeneous communities can harm social capital. Given diverse areas also tend to be high minority-share communities, the result is the problematisation of minority communities in both debates (Kalra and Kapoor, 2009). Demonstrating that diverse neighbourhoods evince high social capital when nested in more integrated wider-communities shows that diversity and social capital are not incompatible; heterogeneous communities can exist with high social capital. It is the intersection of high local diversity and wider-community segregation that can be problematic for social capital. This also challenges the discourse that cultural differences inhibit the emergence of social capital, and the need for policy interventions to reduce such differences. Given diverse neighbourhoods can report just as much social capital as homogeneous neighbourhoods suggests cultural diversity, in and of itself, is not inconsistent with social cohesion. Accordingly, concern would be better focused towards tackling the drivers (or preservers) of segregation to manage any risks of growing diversity for cohesion, as well as recognising the often more detrimental role of disadvantage in neighbourhood social cohesion.

## Abbreviations

OA: Output-Area

LA: Local Authority

ID: Index of Dissimilarity

GHS: General Household Survey

METPAS: Metropolitan Police Public Attitudes Survey

HLM: Hierarchical Linear Models

## Notes

1. This is because the smallest statistical area for which data is available in the Census (the Output-area) is at the ‘street/block’ level. Therefore, we are unable to examine segregation *within* a ‘street/block’.

2. Perceived-threat may respond more to wider-community conditions given labour and housing markets, along with political boundaries, extend across wider-communities.

3. However, we are able to replicate the key findings in both waves independently.

4. We experimented with other segregation indices (e.g. the isolation index). However, the high correlation with diversity made partialling out the relative importance of segregation versus diversity difficult.

5. Given our focus on ethnic diversity we also experimented with Thiel’s multi-group segregation Index. Broadly similar results were returned (unsurprising given its high correlation in our data with the Index of Dissimilarity: *r.* = .89).

6. This is based on rates of violence, burglary, theft and criminal damage, per 1000 at-risk population.

7. Issues could arise given OAs in the GHS contain on average 1.2 individuals, and that a low *n* of level-1 per level-2 units can bias HLM. However, we aim to examine the effect of level-2 measures on level-1 outcomes, and explore cross-level (level-2 and level-3) interactions. Studies suggest maximising the *n* of level-2 units is the most important design for such tests (Bell et al., 2010; Snijders and Bosker, 1999; Wight et al., 2010: 212–213). However, we ran robustness tests (including using the larger, LSOA-level as our level-2 area; or excluding all communities containing only 1 individual; or applying population-averaged models). These returned substantively similar findings. Furthermore, given the higher level-1 *n* per level-2 unit in our London data, replicating our findings within this will also prove a useful robustness test.

8. Identical findings were returned if, amongst White-GB, we applied per cent non-White-GB, not diversity. This is expected given per cent non-White-GB is highly correlated with diversity (*r* = .95).

9. *Coef*: −0.06^+^.

10. For ‘low’ segregated LAs we calculated predicted scores for neighbourhood diversity at Indices of Dissimilarity (ID) of 0.18 (the minimum Local Authority (LA) segregation in our data), 0.2 and 0.3. We then averaged these scores to get the average effect of neighbourhood diversity at ‘low’ segregation. For ‘medium’ segregated LAs we repeated the process but at IDs of .3, .4, .5 and .6. For ‘high’ segregated LAs we repeated the process at IDs of .6, .7, .8 and .82 (the maximum LA segregation in our data).

11. We tested whether this interaction term was driven by differing ethnic compositions between neighbourhoods nested within integrated/segregated wider-communities (e.g. neighbourhoods in segregated wider-communities tend to contain more Pakistanis/Bangladeshis). We rigorously examined this (e.g. by including in our models the proportion of each non-White-GB group). However, in all tests our Model 3 interaction remains strong and significant.

12. The METPAS data allows us to run technically more robust random coefficient multilevel models with around 140 LSOAs per LA.

13. We cannot explore the associations in high segregated wider-communities (> .6 ID) as the highest segregation in London Local Authorities is ID: 0.52.

14. We again experimented with substituting the Index of Dissimilarity for Thiel’s multi-group segregation (*interaction coef*: −0.114*).
